# A Comprehensive Review of Inhaled Nitric Oxide Therapy: Current Trends, Challenges, and Future Directions

**DOI:** 10.7759/cureus.53558

**Published:** 2024-02-04

**Authors:** Divyanshi Kaplish, Jayant D Vagha, Revat J Meshram, Sham Lohiya

**Affiliations:** 1 Pediatrics, Jawaharlal Nehru Medical College, Datta Meghe Institute of Higher Education & Research, Wardha, IND

**Keywords:** precision medicine, challenges and limitations, clinical applications, vasodilatory effects, nitric oxide signaling, inhaled nitric oxide

## Abstract

This comprehensive review explores the multifaceted landscape of inhaled nitric oxide (iNO) therapy, tracing its historical evolution, mechanisms of action, clinical applications, challenges, and future directions. The nitric oxide signaling pathway, characterized by vasodilatory effects and anti-inflammatory properties, forms the foundation of iNO's therapeutic efficacy. Clinical applications are found in neonatal respiratory distress syndrome, pulmonary hypertension, and acute respiratory distress syndrome, showcasing its versatility. However, challenges, including cost considerations, technical intricacies, safety concerns, and resistance, highlight the nuanced landscape surrounding iNO therapy. Implications for clinical practice underscore the need for a tailored and evidence-based approach, considering individual patient characteristics and indications. Recommendations for future research emphasize ongoing exploration, novel indications, and the development of targeted therapies. In conclusion, this review positions iNO as a dynamic and adaptable intervention, poised to reshape therapeutic strategies and enhance patient outcomes in critical care.

## Introduction and background

In recent decades, inhaled nitric oxide (iNO) has emerged as a pivotal therapeutic agent with diverse applications across several medical domains. Nitric oxide, a colorless and diatomic gas, was initially recognized as a vasodilator in the cardiovascular system. However, its inhalation has garnered substantial attention due to its profound impact on pulmonary circulation and its versatile applications in critical care medicine [1]. Nitric oxide exerts pharmacological effects through a complex signaling pathway, influencing vascular tone, inflammation, and oxygenation. Understanding the intricacies of this pathway is fundamental to appreciating the significance of iNO therapy. This section will delve into the historical evolution of iNO as a therapeutic tool, tracing its roots from the discovery of nitric oxide to its present-day applications in neonatology, pulmonology, and beyond [2].

The significance of iNO therapy reverberates across a spectrum of medical conditions, where its unique properties contribute to therapeutic success. Foremost among its applications is the management of neonatal respiratory distress syndrome (RDS), where iNO is pivotal in improving oxygenation and mitigating pulmonary hypertension in preterm infants. Additionally, iNO has found utility in treating pulmonary hypertension, both in pediatric and adult populations, showcasing its versatility in addressing diverse patient demographics [3]. Beyond these primary applications, iNO has been explored in the context of acute respiratory distress syndrome (ARDS) and other conditions characterized by impaired pulmonary function. The ability of iNO to modulate vascular tone and reduce inflammation positions it as a promising therapeutic intervention in scenarios where optimizing gas exchange is paramount [4].

This comprehensive review aims to consolidate the existing knowledge of iNO therapy, providing an in-depth exploration of its current trends, challenges, and future directions. By synthesizing information from historical perspectives to contemporary clinical applications, the review seeks to offer a holistic understanding of the role of iNO in modern medicine. A thorough review becomes imperative as medical practitioners and researchers navigate the evolving landscape of therapeutic options. This article strives to serve as a valuable resource for clinicians, researchers, and healthcare stakeholders by presenting a nuanced perspective on the mechanisms of action, clinical efficacy, challenges, and potential advancements associated with iNO therapy.

## Review

Mechanism of action

Nitric Oxide Signaling Pathway

The therapeutic efficacy of iNO hinges upon its intricate interaction with the nitric oxide signaling pathway, a sophisticated mechanism that holds sway over diverse physiological processes. Nitric oxide, a molecule produced endogenously by various cell types, assumes the role of a potent signaling molecule. In this capacity, it activates the soluble guanylate cyclase (sGC) enzyme, instigating a cascade of events culminating in cyclic guanosine monophosphate (cGMP) synthesis. As a secondary messenger, cGMP orchestrates downstream cellular responses, exerting regulatory control over essential physiological functions [5]. In the context of iNO therapy, introducing exogenous nitric oxide into the respiratory system becomes a pivotal intervention. This introduction serves to amplify and modulate the existing nitric oxide signaling pathway. The inhalation of iNO facilitates a localized and targeted surge in nitric oxide concentrations within the pulmonary environment, subsequently enhancing cGMP production. This augmentation of the signaling cascade assumes paramount importance, forming the mechanistic basis for the vasodilatory and anti-inflammatory effects witnessed in various clinical scenarios [6]. The exogenous application of iNO is that it is a strategic modulator of the nitric oxide signaling pathway, which capitalizes on its inherent regulatory role. This nuanced understanding of the signaling cascade sheds light on the underpinnings of iNO's therapeutic effects, providing a foundation for its diverse applications in conditions such as pulmonary hypertension, neonatal respiratory distress syndrome, and acute respiratory distress syndrome [7].

Vasodilatory Effects and Impact on Pulmonary Circulation

The hallmark feature of iNO lies in its pronounced vasodilatory effect, particularly within the pulmonary vasculature. Upon inhalation, iNO diffuses across the alveolar-capillary membrane, initiating a crucial interaction with hemoglobin and activating sGC in the pulmonary endothelial cells. This activation creates a cascade that ultimately leads to the relaxation of smooth muscle cells, inducing vasodilation within the pulmonary arteries [8]. This selective vasodilatory impact of iNO holds paramount significance in certain medical conditions, notably persistent pulmonary hypertension of the newborn (PPHN) and pulmonary hypertension (PH) in adults. In these scenarios, where constriction of the pulmonary vasculature is a central pathological feature, iNO's ability to induce vasodilation becomes a therapeutic linchpin. By dilating the pulmonary arteries, iNO effectively enhances blood flow and reduces the afterload on the right ventricle. This dual action contributes to improved oxygenation and is a crucial intervention in mitigating pulmonary hypertension [9]. In essence, the targeted vasodilatory effects of iNO within the pulmonary circulation represent a cornerstone in managing conditions characterized by abnormal pulmonary vascular tone. This nuanced mechanism underscores its efficacy in enhancing oxygen delivery and ameliorating the deleterious effects of pulmonary hypertension, ultimately impacting patient outcomes in neonatal and adult populations [10].

Anti-inflammatory Properties

Beyond its vasodilatory effects, iNO stands out for its significant anti-inflammatory properties, adding another layer to its therapeutic utility. Nitric oxide is a signaling molecule within immune cells, intricately modulating inflammatory responses through myriad mechanisms. iNO, as an exogenous source of nitric oxide, plays a crucial role in this modulation, influencing critical aspects of the inflammatory cascade [11]. Research has demonstrated that iNO can effectively inhibit the expression of pro-inflammatory cytokines, impede the activation of adhesion molecules, and curtail the activation of neutrophils. This multifaceted anti-inflammatory action becomes particularly relevant in conditions characterized by pulmonary inflammation, such as ARDS. In these scenarios, where an aberrant inflammatory response exacerbates respiratory compromise, iNO emerges as a targeted therapeutic intervention [12]. By mitigating the inflammatory cascade, iNO addresses the primary cause of respiratory distress and is a preventive measure against further damage to the delicate pulmonary parenchyma. This dual action positions iNO as a modulator of the intricate interplay between inflammation and respiratory compromise, offering a potential avenue for improved outcomes in patients with conditions marked by pulmonary inflammation [4].

Clinical applications

Neonatal RDS

Efficacy of iNO in preterm infants: The efficacy of iNO in preterm infants with severe RDS and pulmonary hypertension has been a topic of debate. A retrospective study evaluated the frequency of PPHN in very preterm infants with severe RDS and the effectiveness of iNO in these patients. The study found that iNO therapy was associated with improved oxygenation in both the PPHN and non-PPHN groups, but it was quicker in the PPHN group. The study concluded that iNO therapy is not recommended for the routine treatment of RDS in premature neonates. Still, in cases of concurrent diagnosis of PPHN, it should be considered carefully [13]. Another drug evaluation reviewed the results of clinical studies on the effects of iNO in preterm infants with RDS and provided contradictory results. Three recent meta-analyses concluded that iNO therapy is not effective in decreasing the risk of death and bronchopulmonary dysplasia (BPD) and cannot be recommended as routine treatment. However, some subgroups of patients, such as infants with PPHN, were suggested for further study [14]. However, it is essential to note that using iNO should be carefully considered based on specific clinical circumstances and in consultation with a neonatal specialist, considering the current evidence and recommendations [15,16].

PH

Use of iNO in pediatric and adult populations: iNO has been indicated for treating PH in pediatric and adult populations. It is known to be effective for patients suffering from acute pulmonary arteriolar constriction, such as pulmonary arterial hypertension (PAH) and PH, due to lung disease and hypoxia. However, its effectiveness may vary in different patient populations [17]. In pediatric patients, iNO has decreased the need for extracorporeal membrane oxygenation (ECMO) support in term and near-term infants [18]. iNO has been studied in pediatric patients undergoing cardiac surgery and has been found effective in lowering pulmonary artery pressure (PAP) and pulmonary vascular resistance (PVR) in the postoperative period [19]. The pulmonary vascular response to iNO has also been studied as part of the assessment for operability in patients with PH associated with congenital heart disease [19].

ARDS

Trials and studies assessing the role of iNO: The role of iNO in managing ARDS has been the subject of extensive investigation through various trials and studies encompassing pediatric and adult populations. The evidence from these studies presents a nuanced perspective on the effectiveness of iNO in improving survival and other clinical outcomes in ARDS patients, yielding mixed results [20]. A comprehensive Cochrane systematic review scrutinizing the impact of iNO on mortality in adults and children with ARDS revealed no statistically significant effect on the longest follow-up mortality. However, a noteworthy finding emerged as the review identified a significant improvement in the PaO2/FiO2 ratio at 24 hours within the iNO group, highlighting a positive impact on oxygenation [20]. This discrepancy between mortality outcomes and oxygenation improvements underscores the complexity of assessing the overall effectiveness of iNO in ARDS. In the context of the ongoing COVID-19 pandemic, a multicenter cohort study focused on critically ill patients with moderate-to-severe ARDS due to COVID-19 suggested a potential role for iNO. The study found that iNO may enhance ventilation, mitigate perfusion imbalances, and reduce pulmonary vascular resistance, thereby relieving hypoxemia associated with ARDS in COVID-19 patients [21]. This observation hints at a specific subset of ARDS cases, such as those induced by COVID-19, where iNO may offer benefits. Scientific societies have acknowledged the complexity of iNO's role in ARDS and issued recommendations. They suggest that iNO might be considered in patients who remain severely hypoxemic despite optimal ventilation and other rescue strategies [22]. These recommendations underscore the cautious and targeted use of iNO as a potential adjunct therapy in specific cases of ARDS where conventional interventions may fall short.

Considerations in patient selection: Using iNO in ARDS patients requires careful consideration of patient selection. Some key considerations include. The severity of hypoxemia and the patient's response to conventional ventilation strategies. The presence of conditions such as PPHN or PH that may benefit from iNO therapy. The potential risks and benefits of iNO therapy, especially in the context of the patient's overall clinical status and comorbidities [23]. Given the mixed evidence regarding the effectiveness of iNO in ARDS, patient selection should be based on a thorough assessment of the individual patient's clinical condition and response to initial management strategies. Close monitoring and regular reevaluation of the patient's response to iNO therapy are essential to determine its ongoing appropriateness [24].

Challenges and limitations

Cost Considerations

Integrating iNO therapy into clinical practice has economic challenges. Cost considerations emerge as a significant factor influencing the adoption of iNO, impacting healthcare institutions, providers, and, ultimately, patient access. The production, storage, and delivery of nitric oxide necessitate specialized infrastructure, contributing to the overall expense associated with this therapeutic intervention. As healthcare systems grapple with budgetary constraints, evaluating the cost-effectiveness of iNO becomes essential for informed decision-making [25]. Moreover, the financial burden extends beyond the direct costs of the gas itself. The requirement for specialized delivery systems and monitoring equipment adds a layer of expense. This economic dimension challenges the universal accessibility of iNO therapy, prompting a critical examination of its cost-effectiveness relative to alternative interventions [26].

Technical Challenges in Administering iNO

The effective administration of iNO demands technical precision that introduces inherent challenges. Maintaining optimal concentrations of iNO in the inspired gas mixture requires sophisticated delivery systems capable of precise dosing. Furthermore, the potential for variability in patient response necessitates continuous monitoring and adjustment, imposing an additional layer of complexity [27]. Technical challenges extend beyond delivery systems, including gas purity, storage, and equipment maintenance issues. Ensuring a consistent and reliable supply of high-quality nitric oxide is paramount to the success of iNO therapy. These technical intricacies underscore the need for specialized training among healthcare professionals administering iNO, adding to the logistical challenges associated with its implementation [28].

Potential Adverse Effects and Safety Concerns

While iNO offers therapeutic benefits, its application is not devoid of potential adverse effects and safety concerns. The most frequently reported adverse event is methemoglobinemia, a condition characterized by an abnormal increase in methemoglobin levels, which can impair oxygen delivery. Rigorous monitoring of methemoglobin levels is essential to prevent this complication [29]. Moreover, the interaction of nitric oxide with oxygen may form nitrogen dioxide, a toxic byproduct. Stringent safety measures, including proper ventilation and scavenging systems, are imperative to mitigate the risk of nitrogen dioxide exposure. Additionally, concerns about the long-term effects of iNO therapy, particularly in vulnerable populations such as neonates, warrant ongoing research and surveillance [30].

Resistance to iNO Therapy

The phenomenon of resistance poses an intriguing challenge within the realm of iNO therapy, as certain patients manifest limited or no response to the vasodilatory effects of nitric oxide. The mechanisms contributing to this resistance are complex and multifactorial, encompassing various facets such as impaired bioavailability of nitric oxide, alterations in downstream signaling pathways, and potentially underlying genetic factors [31]. This enigma of resistance necessitates a thorough exploration to unravel its intricacies and enhance the efficacy of iNO as a therapeutic modality. Understanding and overcoming resistance to iNO therapy are critical for optimizing patient outcomes, especially in conditions where iNO is considered a cornerstone intervention. The multifaceted nature of resistance prompts researchers and clinicians to delve into comprehensive strategies. One avenue involves identifying biomarkers or predictive indicators to discern the likelihood of a patient's responsiveness to iNO. By deciphering these markers, healthcare providers may be better equipped to tailor treatment strategies and allocate resources more judiciously [32]. Refining dosing strategies represents another crucial facet of addressing resistance to iNO. Tailoring the dosage to individual patient needs, guided by age, weight, and specific disease characteristics, can enhance therapeutic efficacy. Additionally, exploring combination therapies that complement the vasodilatory effects of iNO may offer a synergistic approach to overcome resistance. This avenue of research involves investigating the interplay between iNO and other therapeutic agents to potentiate their collective impact [31].

Current trends

Recent Advancements in iNO Delivery Systems

The iNO delivery system market has been experiencing growth, driven by the increasing prevalence of respiratory disorders and technical advancements in iNO delivery systems. The hypoxemic respiratory failure treatment segment accounted for the most significant global market share in 2021, indicating the widespread use of iNO delivery systems for this application [33]. The market is bifurcated into systems and disposables, with the disposables product segment accounting for the largest revenue share in 2020. This growth is attributed to the increased demand for nebulizer masks in hospitals and the low cost of raw materials [34]. Technical developments in iNO delivery systems are projected to expedite market expansion by creating portable, user-friendly devices that provide patients with accurate iNO delivery [35].

Novel Applications and Off-Label Use

In addition to its established use in conditions such as PAH and PPHN, iNO has been the subject of novel applications and off-label use. In-vitro studies suggest that iNO is effective in suppressing the severe acute respiratory syndrome coronavirus 2 (SARS-CoV-2) virus, and it has been used in treating ARDS, a characteristic of COVID-19-positive patients [35]. iNO is proposed to treat conditions associated with reversible pulmonary vasoconstriction and pulmonary vasoconstriction, indicating its potential in a broader range of respiratory conditions [35]. Table 1 outlines novel applications and uses of off-label iNO.

**Table 1:** Outlining novel applications and off-label uses of inhaled nitric oxide (iNO)

Application	Description
Neuroprotection in ischemic stroke [36]	Ongoing research explores the potential of iNO as a neuroprotective agent in ischemic stroke. Preclinical studies have shown promising results in reducing neuronal damage and improving neurological outcomes. Clinical trials are underway to validate these findings in human subjects.
Sepsis-associated acute respiratory distress syndrome (ARDS) [24]	iNO is being investigated as an adjunct therapy in ARDS associated with sepsis. Preliminary studies suggest that iNO may mitigate inflammation and improve oxygenation in septic ARDS, though further research is needed to establish its efficacy and safety in this context.
Cardiovascular diseases [37]	Emerging studies explore the role of iNO in cardiovascular diseases, including heart failure and ischemic heart disease. The vasodilatory effects of iNO may have potential benefits in optimizing cardiac function and improving outcomes in certain cardiovascular conditions.
Neurodegenerative disorders [38]	The neuroprotective properties of nitric oxide raise interest in exploring iNO as a potential therapy for neurodegenerative disorders such as Alzheimer's and Parkinson's disease. Preclinical studies indicate neuroprotective effects, but its still too early for clinical validation.
Infectious diseases [39]	Research is underway to investigate the antimicrobial properties of iNO in treating infections, particularly respiratory infections. iNO's potential to inhibit the growth of bacteria and viruses in the respiratory tract may have implications for treating infectious diseases.

Integration of iNO in the Era of Precision Medicine

Integrating iNO into the era of precision medicine represents a realm of ongoing development, holding the promise of personalized therapeutic interventions tailored to individual patient characteristics and specific disease states. Notably, iNO has gained approval from the US FDA for use in newborns with PPHN. In this context, iNO has demonstrated efficacy in reducing the need for ECMO, showcasing its potential as a targeted and effective intervention for a specific neonatal population [40]. Furthermore, the application of iNO extends beyond its established use in PPHN. The routine utilization of acute vasoreactivity testing, predominantly employing iNO, in the catheterization laboratory has become integral for diagnosing and managing PAH in pediatric patients [40]. This strategic use of iNO exemplifies its role in guiding therapeutic decisions with precision, particularly in a population where tailored treatment approaches are paramount.

The landscape of iNO is further evolving with advancements in delivery systems and expanding applications. These developments enhance the versatility of iNO and accentuate its potential for integration into the framework of precision medicine. The ongoing refinement of delivery systems, coupled with a growing understanding of the nuances in patient responses, underscores the trajectory toward more individualized and targeted iNO therapy. As precision medicine continues to shape the future of healthcare, the dynamic role of iNO in neonatal care and pulmonary hypertension exemplifies its potential as a paradigm for personalized therapeutic strategies. This synergy between evolving technologies and expanding clinical applications positions iNO as a critical player in pursuing precision medicine's goals of optimized patient outcomes through tailored and nuanced interventions [40].

Future directions

Ongoing Research and Clinical Trials

The landscape of iNO therapy is dynamic, with ongoing research and clinical trials contributing to our evolving understanding of its potential applications and optimizing its use. These studies explore novel aspects of iNO, from refining dosing protocols to investigating its efficacy in emerging medical conditions. Tracking these endeavors is crucial for staying abreast of the latest developments and breakthroughs [41]. Recent trials have delved into using iNO in conditions beyond traditional applications, such as neuroprotection in ischemic stroke or as an adjunct therapy in sepsis-associated ARDS. These avenues of exploration hold promise for expanding the therapeutic repertoire of iNO and may herald new frontiers in its clinical application [42].

Potential Expansion of iNO Therapy to New Indications

The potential expansion of iNO therapy to new indications marks a captivating frontier as our comprehension of nitric oxide's physiological effects advances. Ongoing investigations are actively exploring the applicability of iNO in previously uncharted medical territories, offering a promising avenue for diversifying its clinical utility. The exploration of iNO in cardiovascular medicine is particularly intriguing, where burgeoning interest surrounds its potential to address conditions such as heart failure and ischemic heart disease. Leveraging its well-documented vasodilatory effects and the suggested cardioprotective properties of nitric oxide, researchers are delving into the prospect of iNO as a therapeutic intervention in the intricate landscape of cardiovascular disorders [43].

Simultaneously, the neuroprotective effects of nitric oxide are emerging as a focal point in the exploration of iNO therapy. The potential application of iNO in neurodegenerative disorders adds a new layer of therapeutic possibilities, extending beyond the traditional realms of pulmonary and circulatory medicine. The quest to harness the neuroprotective properties of nitric oxide signifies an innovative stride toward addressing conditions that have long posed significant challenges in the field of neurology [44]. As these investigations unfold, the potential expansion of iNO therapy into cardiovascular, neurological, and infectious disease realms broadens the scope of its clinical applications. It underscores the versatility of nitric oxide as a multifaceted signaling molecule. These developments hold promise for the future, where iNO may play a pivotal role in reshaping therapeutic approaches across diverse medical landscapes, offering new avenues for improved patient outcomes and innovative treatment modalities [5].

Development of Targeted Therapies Based on Individual Patient Characteristics

Advancements in precision medicine have sparked interest in tailoring therapeutic interventions based on individual patient characteristics, and iNO therapy is no exception. As we delve into the era of personalized medicine, there is a growing recognition of the heterogeneity in patient responses to iNO. Developing targeted therapies involves identifying biomarkers or phenotypic characteristics that can predict patient responsiveness [45]. Understanding the genetic, molecular, or physiological factors influencing a patient's response to iNO is critical to optimizing its therapeutic benefits. This personalized approach not only enhances the efficacy of iNO but also minimizes the risk of adverse effects and improves overall patient outcomes. Integrating cutting-edge technologies, such as genomics and biomarker profiling, into clinical decision-making processes is a promising avenue for realizing this vision [46].

Regulatory and ethical considerations

Approval and Regulation of iNO as a Therapeutic Agent

The approval and regulation of iNO as a therapeutic agent falls under the jurisdiction of regulatory agencies such as the FDA in the United States and the European Medicines Agency (EMA) in the European Union. iNO is approved for specific indications, such as the treatment of term and near-term infants with hypoxic respiratory failure associated with clinical or echocardiographic evidence of pulmonary hypertension and for the treatment of PPHN [18]. The regulatory process for iNO involves submitting clinical data demonstrating its safety and efficacy for the proposed indications. The regulatory agencies review the data to determine whether the drug's benefits outweigh its risks and whether it can be approved for use in the target patient population.

Ethical Considerations in the Use of iNO, Especially in Research Settings

In research settings, using iNO is subject to ethical considerations regarding protecting human subjects and conducting research according to ethical principles and guidelines. Researchers must obtain informed consent from study participants, ensure the confidentiality of participant data, and minimize the risks of harm to participants [47]. Using iNO in research should also adhere to beneficence, non-maleficence, and respect for people. This includes ensuring that the potential benefits of the research outweigh the risks, minimizing the risks of harm to participants, and respecting the autonomy and rights of participants [48]. The approval and regulation of iNO as a therapeutic agent are overseen by regulatory agencies, and its use in research settings is subject to ethical considerations related to the protection of human subjects and the conduct of research by ethical principles and guidelines [49].

## Conclusions

In conclusion, the exploration of iNO therapy through this comprehensive review reveals its dynamic role in modern medicine. The historical journey from the discovery of nitric oxide to its current applications underscores its pivotal position in addressing various medical conditions. The intricate mechanisms of action, characterized by the nitric oxide signaling pathway, vasodilatory effects, and anti-inflammatory properties, provide a robust foundation for understanding its therapeutic impact. Clinical applications, spanning neonatology to pulmonology, showcase the versatility of iNO across a spectrum of disorders. However, the review also unveils challenges, including economic considerations, technical intricacies, safety concerns, and the intriguing resistance phenomenon. These findings have profound implications for clinical practice, urging practitioners to balance the potential benefits of iNO against its challenges while emphasizing individualized, evidence-based approaches. Looking forward, recommendations for future research underscore the importance of ongoing exploration, novel indications, and the development of targeted therapies based on individual patient characteristics. In the ever-evolving landscape of medicine, inhaled nitric oxide emerges as a promising, adaptable intervention with the potential to reshape therapeutic strategies and enhance patient outcomes.
